# The agger nasi approach to the uncinate process: From top to bottom

**DOI:** 10.1016/j.bjorl.2025.101675

**Published:** 2025-07-29

**Authors:** Miguel Soares Tepedino, Luziana de Lima Ramalho, Leonardo Balsalobre, Andrea Santos Dumont Costa Curta, Debora de Carvalho Garcez, Rogerio Pezato

**Affiliations:** a Universidade do Estado do Rio de Janeiro, Departamento de Otorrinolaringologia e Cirurgia de Cabeça e Pescoço e Base do Crânio, Rio de Janeiro, RJ, Brazil; b Policlínica de Botafogo, Departamento de Otorrinolaringologia e Cirurgia de Cabeça e Pescoço e Base do Crânio, Rio de Janeiro, RJ, Brazil; c Universidade Federal de São Paulo, Departamento de Otorrinolaringologia e Cirurgia de Cabeça e Pescoço e Base do Crânio, São Paulo, SP, Brazil; d Rede D’Or São Luiz, Departamento de Radiologia, Rio de Janeiro, RJ, Brazil; e Fleury Medicina e Diagnóstico, Rio de Janeiro, RJ, Brazil; f Universidade do Estado do Rio de Janeiro, Departamento de Radiologia, Rio de Janeiro, Brazil

**Keywords:** Uncinectomy, Uncinate process, Agger nasi, Endoscopic sinus surgery

## Abstract

**Objective:**

To describe a novel endoscopic approach to the Uncinate Process (UP) via the agger nasi region and evaluate its anatomical basis and clinical outcomes.

**Methods:**

This study comprised two components. In the imaging study, 51 paranasal sinus CT scans were analyzed to measure the distance between the UP and the medial orbital wall at the agger nasi and ethmoid infundibulum regions, using standardized coronal and axial planes. In the clinical study, 53 patients with chronic rhinosinusitis underwent uncinectomy through the agger nasi approach. Pre- and postoperative SNOT-22 scores were compared after 6-months of follow-up, and complications were recorded.

**Results:**

The distance from the UP to the orbit was significantly greater at the agger nasi region compared to the ethmoid infundibulum on both sides (p < 0.001). Clinically, the agger nasi approach allowed consistent identification of the maxillary sinus ostium and preservation of key anatomical structures. There was a significant improvement in SNOT-22 scores postoperatively (p < 0.001), with no major complications observed.

**Conclusion:**

The agger nasi approach to the UP is a safe, reproducible, and effective technique. The greater distance from the UP to the orbit in this region may reduce the risk of orbital injury and facilitate complete identification of the natural drainage pathway of the maxillary sinus.

**Level of evidence:**

3.

## Introduction

Uncinectomy is the first step in sinus surgery. Over the last 30-years, Endoscopic Sinus Surgery (ESS) has become a major treatment option for many sinonasal diseases, especially chronic rhinosinusitis, a condition in which the success rate of ESS ranges from 76% to 98%.^1^, ^2^, ^3^, ^4^ Several seemingly safe uncinectomy techniques have been published over the years, with those described by Stammberger and Wormald et al being most widely accepted and employed.^5^, ^6^, ^7^, ^8^, ^9^

Although studies reveal that the major uncinectomy techniques are generally well accepted by surgeons, ESS is still associated with a high rate of revision surgery (estimated at 10%–17%).^10^, ^11^ Incomplete ethmoidectomy and, more specifically, the presence of remnants of the Uncinate Process (UP) are among the leading causes of revision ESS. There is also a high incidence of postoperative recirculation of mucus, an iatrogenic condition caused by failure to correctly identify the primary ostium of the maxillary sinus after uncinectomy. Uncinectomy has the potential for several complications, such as orbital penetration, exposure of orbital fat, orbital edema, nasolacrimal duct injury, and epiphora.^3^, ^9^, ^12^, ^13^

In this context, we present an option of a novel surgical approach to the uncinate process, as well as its anatomic and radiologic rationale and postoperative results.

## Methods

Uncinectomy was performed by the agger nasi technique as described below. For demonstration purposes, this was done on a cadaver model. This is a prospective study and was divided into two parts: imaging and in vivo. All participants provided written informed consent. The study was approved by the local Research Ethics Committee with number 79548324.8.0000.5259.

### Imaging study

**Fig. 1:**
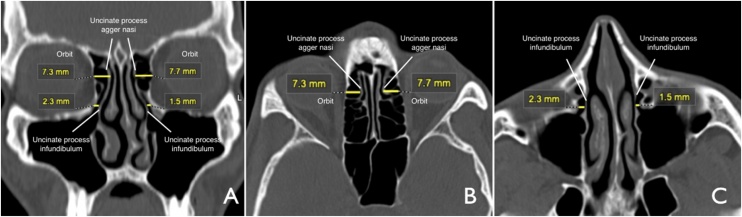
Computed tomography of the paranasal sinuses (bone window). Horizontal yellow lines measure the greatest distance from the uncinate process to the medial wall of the orbit at the level of the agger nasi and ethmoid infundibulum. A: coronal slice.

The Computed Tomography (CT) scans of the paranasal sinuses in patients with or without sinonasal disease, where it was possible to analyze the entire extent of the uncinate process, were analyzed. The uncinate process was identified in the region of the ethmoid infundibulum and in the agger nasi. A horizontal line was drawn in the coronal and axial planes and the greatest distance between the uncinate process, and the medial wall of the orbit was measured in the region of the agger nasi and the ethmoid infundibulum. These measurements were obtained on the right and left sides. All scans were assessed by the same radiologist (Fig. 1).

The inclusion criteria for the imaging study were age >18-years, preserved sinonasal anatomy, and no history of surgical procedures or diseases that might have altered sinonasal anatomy, such as benign or malignant tumors and trauma.

### In vivo study

We assessed the impact of the agger nasi approach to the uncinate process on the quality of life in patients with chronic rhinosinusitis with or without polyps by comparing SNOT-22 scores before and 6-months after procedures performed using this technique.^14^ Major surgical complications at 6-months of follow-up were also recorded.

The inclusion criteria for the in vivo study were age >18-years, a diagnosis of CRS (with an indication for endoscopic endonasal sinus surgery that included the need for removal of the uncinate process) according to EPOS-2020 criteria, and prior classification according to endotype and phenotype. Patients with previous sinonasal surgery and those in whom it was not possible to identify the uncinate process on surgery were excluded. Data were collected from January 2023 to January 2024.

### Surgical technique

**Fig. 2:**
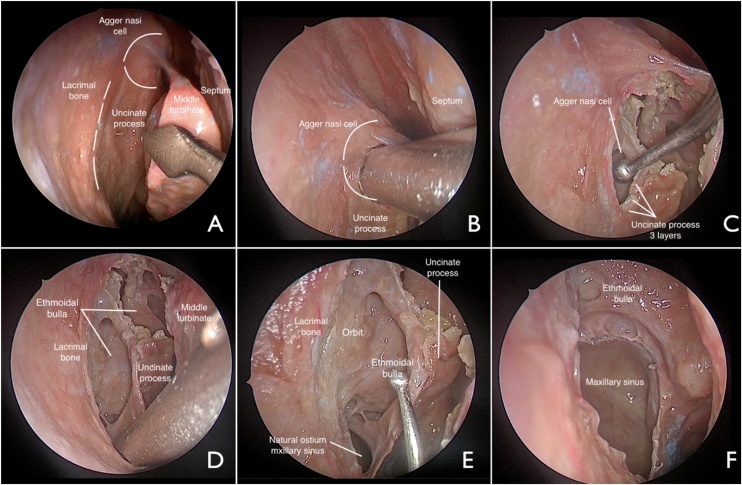
Demonstration of uncinectomy using the agger nasi technique. Endonasal dissection of the right nasal fossa (45-degree scope view). A: middle turbinate medialization and exposure of the middle meatus. B: horizontal incision of the uncinate process.

**Fig. 3:**
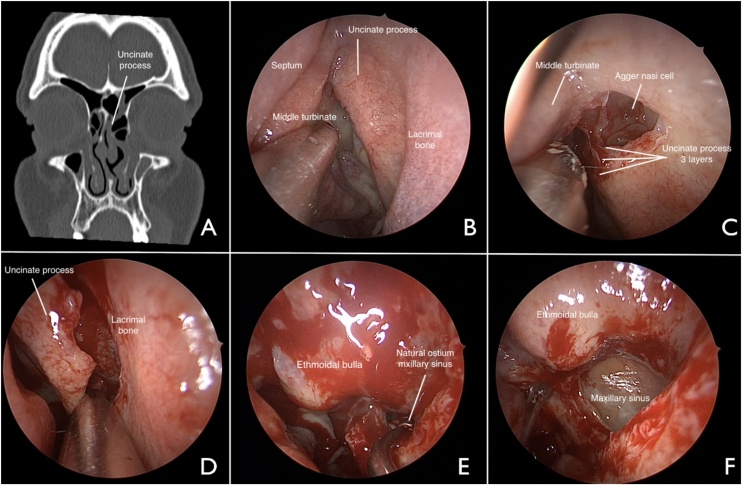
Uncinectomy using the agger nasi technique in a patient with chronic rhinosinusitis. A: computed tomography of paranasal sinuses (bone window, coronal slice). Uncinate process visible on the left side. B-F: endonasal approach to the left nasal fossa (45-degree scope view). B: exposure of the middle meatus. C: horizontal incision with shaver. C: identification of the 3 layers of the uncinate process (medial mucosa, bone, lateral mucosa). D: detachment of the uncinate process from the lacrimal bone anteriorly and inferiorly. E: identification of the natural drainage ostium of the maxillary sinus. F: final appearance of maxillary antrostomy after complete resection of the uncinate process.

The procedure was always performed by the same surgeon following the same steps, regardless of the underlying diagnosis. In brief, through a 4-mm, 45-degree Karl Storz endoscope, the UP was inspected with the aid of a seeker. Resection of the UP began with the use of a 40-degree shaver and Medtronic handpiece. The medial wall of the agger nasi was removed with a horizontal incision from posterior to anterior; the orbit, layers of the uncinate process, and attachment of the lower remnant of the UP into the lacrimal bone (lateral wall of the nose) were identified. Again, with the aid of the shaver, the UP was separated from the lacrimal bone, from superior to inferior, at the limit of its attachment between the two bones, thus exposing the entire ethmoid infundibulum. Once the inferior portion of the lacrimal bone was reached, the lower remnant of the UP was detached from the posterior fontanelle using the shaver or micro scissors (Fig. 2, Fig. 3). In patients with sinonasal polyposis, before starting the incision of the uncinate process, the polyps were resected with a microdebrider, thus the uncinate process was identified both in the agger nasi region and in the infundibulum region, furthermore the middle turbinate was resected if affected by the disease. When the middle turbinate was preserved, a suture (4‒0 Vicryl) was placed between the turbinates and the nasal septum. The microdebrider was only introduced after direct identification of anatomical landmarks, and its use was under constant endoscopic visualization with a 45-degree endoscope.

All patients were followed up postoperatively with outpatient nasal endoscopy using a 30-degree Karl Storz optic. Specifically, patients were examined for synechiae between the middle turbinate and lateral wall and between the middle turbinate and nasal septum, presence of epiphora, mucus recirculation, or pathological stenosis of the maxillary antrostomy.

### Statistical analysis

The Shapiro-Wilk test was used to evaluate the normality of distribution, and the nonparametric Mann–Whitney test was then applied to compare the distances of the landmarks measured on the sinus CT scans. The nonparametric Wilcoxon test was used to compare pre- vs. postoperative SNOT-22 scores. The significance level was set at p < 0.05. The sample size calculation was carried out considering the adult population of Rio de Janeiro around 4.000.000 inhabitants, and adopting a sampling error of 10% and a confidence interval of 90%, the suggested sample was at least 44 participants, our study adopted 51 patients using bilateral CT scan measures, totaling 102 records. Furthermore, we include 53 surgical patients who underwent ESS, 46 underwent bilateral surgery.

## Results

### Imaging study

Fifty-one Computed Tomography (CT) scans of the paranasal sinuses were analyzed. All patients had agger nasi cells. Participants in the imaging study had an average age of 57 years (range, 18–97 years); there were 27 women (53%) and 24 men (47%). The measurements of the horizontal line were obtained on the right and left sides, for a total of 102 measurements in the agger nasi region and 102 measurements in the infundibulum region (Table 1).

**Table 1:** Distance from the unciform process to the medial wall of the orbit on CT: at the level of agger nasi and at the level of infundibulum.

	Right agger nasi (mm)	Left agger nasi (mm)	Right infundibulum (mm)	Left infundibulum (mm)
Mean	6.82	7.31	3.15	3.12
Standard deviation	1.40	1.74	1.76	1.99
Minimum	3.67	3.24	0.95	1.34
Maximum	10.17	10.04	7.82	8.93

The distance from the uncinate process to orbit in the infundibulum region is so small that it measured 0.95 millimeters on one side, while in the agger nasi region the smallest distance was 3.24 mm. The overall distance from the UP to the medial wall of the orbit at the agger nasi was significantly greater in men (p = 0.019), but there was no gender difference in the distance from the ethmoid infundibulum to the medial wall of the orbit (p = 0.564).

**Fig. 4:**
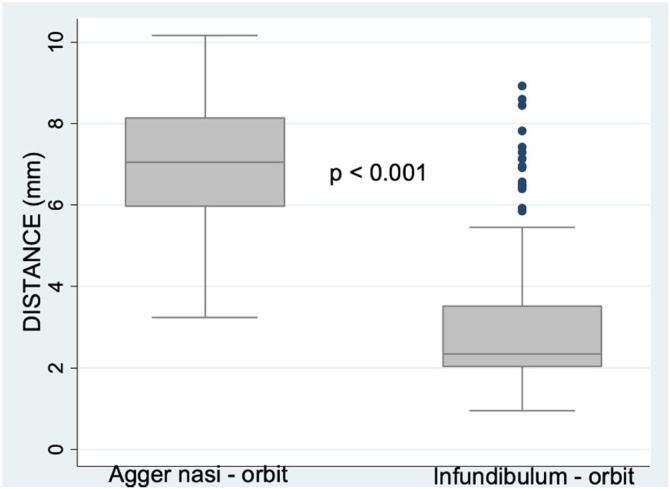
Distribution of measurements of distance between the uncinate process and the medial wall of the orbit at the agger nasi and ethmoid infundibulum.

The overall distance from the UP to the medial wall of the orbit at the agger nasi was greater than the distance from the UP to the medial wall of the orbit at the ethmoid infundibulum on both right and left sides, with statistically significant differences found independently (p < 0.001 for both sides). These comparisons were conducted separately to ensure statistical independence (Fig. 4).

### Surgical patients

We selected 53 patients who underwent ESS, 46 underwent bilateral surgery, 26 of whom were diagnosed with diffuse CRS, not type 2, without Nasal Polyps (CRSsNP), and 20 patients with CRS, type 2, with Nasal Polyps (CRSwNP). Surgery was unilateral in 7 patients diagnosed with CRS secondary to maxillary fungal ball, for a total of 99 uncinectomies. The middle turbinate was resected bilaterally in 9 patients with CRSwNP. Among these, 20 patients (37.7%) had CRSwNP. In these cases, polyps were resected in the initial phase to allow clear visualization of the uncinate process, which was then approached using the same agger nasi technique.

The average age of the patients was 47-years (range 18–83 years); 23 were female (43.4%) and 30 male (56.6%).

**Fig. 5:**
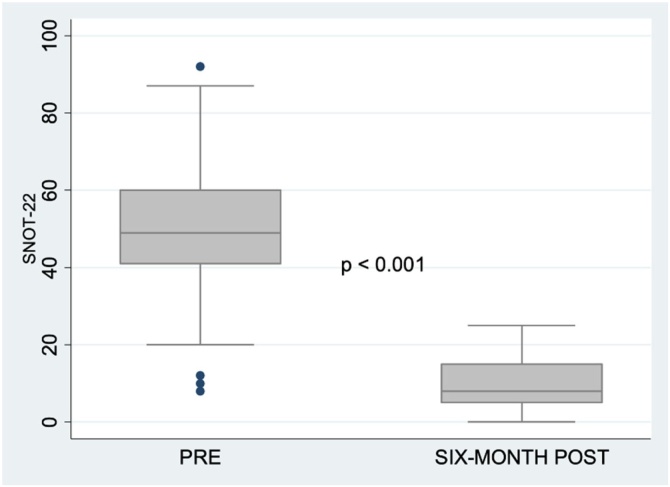
Distribution of pre- and 6-month postoperative SNOT-22 scores.

There was a significant reduction in SNOT-22 scores postoperatively (p < 0.001) (Fig. 5). No patient had nasolacrimal duct exposure or injury, nor was orbital fat exposure observed during surgery. There were no cases of epiphora, synechiae of the lateral wall to the nasal septum, mucus recirculation, or pathological stenosis of the maxillary antrostomy during the 6-month postoperative period. Two patients (2%) had synechiae form between the nasal septum and the middle turbinate unilaterally.

## Discussion

The prevalence of agger nasi cells has ranged from 81.8% in a study conducted in Taiwan to 98.5% in a study conducted in the U.S., which is representative of most cases, even across different populations.^15^, ^16^ The extent of pneumatization also differs among patients, as the anatomy of the ethmoid is extremely variable. The imaging portion of our study demonstrated that the distance from the UP to the orbit in the agger nasi region is consistently and significantly greater than the same distance in the ethmoid infundibulum. In this sense, starting the UP incision at the infundibulum, as described by Stammberger and Wormald et al., there would be a greater risk of injury to the medial wall of the orbit due to the shorter distance. In our study, the overall distance from the UP to the medial wall of the orbit at the agger nasi was greater than the distance from the UP to the medial wall of the orbit at the ethmoid infundibulum, this difference was statistically significant. It was also observed that on one side the distance from the uncinate process to orbit in the infundibulum region is so small that it measured 0.95 millimeters, while in the agger nasi region the smallest distance was 3.24 mm. It bears stressing that the advantages of this new approach that we present also include the ease of identifying the three layers of the UP (lateral mucosa, bone, and medial mucosa) as well as exposing the anterior attachment of the UP and resecting it completely, which ensures identification of the natural drainage ostium of the maxillary sinus.

The presence of UP remnants is one of the most frequent findings in patients with CRS undergoing ESS, occurring in 37%–60% of cases.^3^, ^17^, ^18^, ^19^ Even with progress in surgical techniques and technology, imaging findings in revision surgery have remained strikingly similar over the last 30-years. This raises an important question regarding the outcomes of commonly used operative techniques. It is interesting to note that, although many surgeons are satisfied with their current uncinectomy techniques, this satisfaction may not correspond to the effectiveness of the procedure, since a substantial number of patients ultimately require revision surgery.

Several studies have reported that ethmoid remnants and postoperative recirculation of the maxillary sinus are among the main findings in patients who undergo revision surgery.^1^, ^3^, ^5^, ^6^^,^^17^, ^18^, ^19^ Identification of the natural ostium of the maxillary sinus and its inclusion in the middle maxillary antrostomy is crucial to avoid the phenomenon of mucus recirculation. With the top-down agger nasi approach uncinectomy described herein, all natural drainage ostia were identified. In the present study, after UP resection, a maxillary antrostomy was performed from anterior (i.e., from the natural ostium) to posterior, with resection of the posterior fontanel all the way to the posterior wall of the maxillary sinus. No cases of postoperative recirculation were observed at 6-months of outpatient follow-up.

Nasolacrimal duct injury is also a concern during uncinectomy, especially when performed with a backbiter in retrograde fashion. Wormald et al. reported exposure of the nasolacrimal duct in only 1 patient out of 636 procedures. With our technique, there were no cases of duct exposure or injury. We believe that surgical exposure of the UP and the ease of identifying its exact attachment to the lacrimal bone greatly reduce any risk of iatrogenic injury in this region.

Another relevant finding from our sample was the statistically significant decrease in SNOT-22 scores 6-months after surgery (p < 0.001), which reflects a major improvement in patients’ quality of life during postoperative follow-up. From the point of view of operative technique, 44 of the 53 patients had the middle turbinate spared and underwent suture conchopexy to the septum. In two patients, which represents 2% of the total, unilateral synechiae were observed between the middle turbinate and the nasal septum. This had no impact on quality of life; both patients had SNOT-22 scores < 10, which are considered normal. Furthermore, middle turbinate medialization is considered by many authors to be beneficial to the patient.^20^

This is an initial study with a limited number of cases. The approach presented uses a microdebrider, which is a tool that requires training to be used safely. This is an alternative to traditional techniques. Further publications are needed to confirm the findings presented.

## Conclusion

In this study, the agger nasi approach to the uncinate process demonstrated feasibility and safety. Based on our radiologic findings, the distance from the UP to the orbit in the agger nasi region is consistently, significantly greater than the same distance in the region of the ethmoid infundibulum, which may reduce the risk of complications. Clinically, the technique allowed adequate identification of the uncinate process and natural maxillary ostium in both CRSsNP and CRSwNP patients. While not intended to replace conventional uncinectomy techniques, this method may serve as a complementary approach.

## ORCID ID

Luziana de Lima Ramalho: 0009-0002-3014-1237

Leonardo Balsalobre: 0000-0001-8251-9217

Debora de Carvalho Garcez: 0000-0002-9756-3314

Rogerio Pezato: 0000-0002-9813-4466

## Funding

All participants provided written informed consent. The study was approved by the local Research Ethics Committee with number 79548324.8.0000.5259 in 2023.

All study data are available for verification.

## Declaration of competing interest

The authors declare no conflicts of interest.
